# Campylobacter fetus Adrenal Abscess

**DOI:** 10.7759/cureus.73556

**Published:** 2024-11-12

**Authors:** Jack W Kerferd, Aidan R McLachlan, Heeral Thakkar, Samuel W Baumgart, Genevieve McKew

**Affiliations:** 1 Department of Microbiology and Infectious Diseases, Concord Repatriation General Hospital, Sydney, AUS; 2 Faculty of Medicine and Health, University of Sydney, Sydney, AUS; 3 Sydney Infectious Diseases Institute, University of Sydney, Sydney, AUS

**Keywords:** abdominal sepsis, adrenal, adrenal abscess, campylobacter fetus, campylobacter fetus subspecies fetus (c fetus)

## Abstract

A 69-year-old highly comorbid female patient presented to the emergency department with sepsis following a month of fevers, myalgias, and lethargy. Abdominal imaging revealed an adrenal abscess, an aspirate of which grew *Campylobacter fetus (C. fetus*). The patient was treated with meropenem and then azithromycin; however secondary infection of the abscess cavity with an extended-spectrum beta-lactamase (ESBL)-producing *Escherichia coli *(*E. coli*) and failure of source control led to an extended clinical course. This case highlights the only documented case of *C. fetus *adrenal abscess, and a literature review of *C. fetus*-associated abscess presentations is provided.

## Introduction

Campylobacteriosis encompasses the diseases caused by microaerophilic Gram-negative bacteria of the genus *Campylobacter*. *Campylobacter jejuni *(*C. jejuni* ) and *Campylobacter coli* (​​​​​​*C. coli*) are most commonly isolated from patients with diarrheal illnesses. While less common overall, *Campylobacter ​​​​​​​fetus* (*C. fetus*) is an important human pathogen. *C. fetus *is a non-spore-forming, Gram-negative rod that is classically curved or spiral-shaped. It is catalase-positive and motile through a single unipolar or bipolar flagellum. Bacteremic patients may present with a nonspecific acute to subacute febrile illness [1]. However, complications can occur from dissemination including endovascular infection, mycotic aneurysms with thrombosis, and rarely, abscess formation. Here, we present a rare case of an adrenal abscess due to *C. fetus* infection.

## Case presentation

A 69-year-old female patient presented to the emergency department with a one-month history of fevers, lethargy, myalgias, nausea, vomiting, and intermittent loose stools. She had a history of schizoaffective disorder, recurrent urinary tract infections, obstructive sleep apnea with pulmonary hypertension, diabetes, and obesity (body mass index (BMI), 64.8 kg/m^2^). At presentation, the patient was drowsy, tachypneic (respiratory rate, 40 breaths per minute), and tachycardic (heart rate, 117 beats per minute). Respiratory and abdominal examinations were limited by body habitus and did not suggest clear localizing pathology. Initial laboratory investigations (Table 1) demonstrated profound metabolic acidosis (pH, 7.10) and acute kidney injury (creatinine of 243 µmol/L compared to baseline creatinine of 64 µmol/L). Inflammatory markers, including neutrophil count and C-reactive protein (CRP) were significantly elevated at 15.2 x 109/L and 423 mg/L, respectively. Initial chest X-ray was consistent with pulmonary edema and did not show focal consolidation. Urine culture grew *Escherichia coli* (*E. coli*) with >10^8^ colony-forming units/L (CFU/L). This was sensitive to ampicillin, cefalexin, trimethoprim, gentamicin, and nitrofurantoin. A screening multiresistant organism rectal swab isolated an extended-spectrum beta-lactamase (ESBL)-producing *E. coli *suggesting colonization*.* Initial blood cultures grew *Propionibacterium lymphophilum* in a single anaerobic bottle, favored to represent a contaminant in light of subsequent sterile blood cultures. Empirical management for urosepsis and decompensated heart failure was commenced, including antimicrobial therapy with ceftriaxone, gentamicin, and clindamycin. However, she acutely deteriorated while in the emergency department and developed hypotension, hypoxia, and worsening acid-base balance requiring intubation and multiple vasopressor support in the intensive care unit (ICU).

**Table 1:** Summary of patient's laboratory data on admission EGFR: Estimated glomerular filtration rate; GGT: gamma-glutamyl transferase; ALP: alkaline phosphatase; ALT: alanine aminotransferase; AST: aspartate aminotransferase; CRP: C-reactive protein

Test	Result	Reference range
White blood cell count	18.5 x 10^9^/L	4.0-10.0 x 10^9^/L
Neutrophils	15.2 x 10^9^/L	2.0-7.0 x 10^9^/L
Lymphocytes	2.5 x 10^9^/L	1.0-3.0 x 10^9^/L
Monocytes	0.8 x 10^9^/L	0.2-1.0 x 10^9^/L
Eosinophils	0.1 x 10^9^/L	0.0-0.5 x 10^9^/L
Basophils	0.0 x 10^9^/L	0.0-0.1 x 10^9^/L
Hemoglobin	80 g/L	120-150 g/L
Platelet count	518 x 10^9^/L	150-400 x 10^9^/L
Urea	25.8 mmol/L	3.5-8.0 mmol/L
Creatinine	243 µmol/L	45-90 µmol/L
EGFR	17 mL/min/1.73m^2^	≥60 mL/min/1.73m^2^
Sodium	132 mmol/L	135-145 mmol/L
Potassium	5.0 mmol/L	3.5-5.2 mmol/L
Bilirubin	10 µmol/L	≤20 µmol/L
GGT	310 U/L	5-35 U/L
ALP	187 U/L	30-110 U/L
ALT	14 U/L	10-35 U/L
AST	23 U/L	10-35 U/L
pH	7.10	7.30-7.40
Venous pCO2	38 mmHg	40-50 mmHg
Bicarbonate	12 mmol/L	22-32 mmol/L
Lactate	2.3 mmol/L	≤1.6 mmol/L
Base excess	-17 mmol/L	-3-3 mmol/L
CRP	423.0 mg/L	≤4.9 mg/L

**Figure 1:**
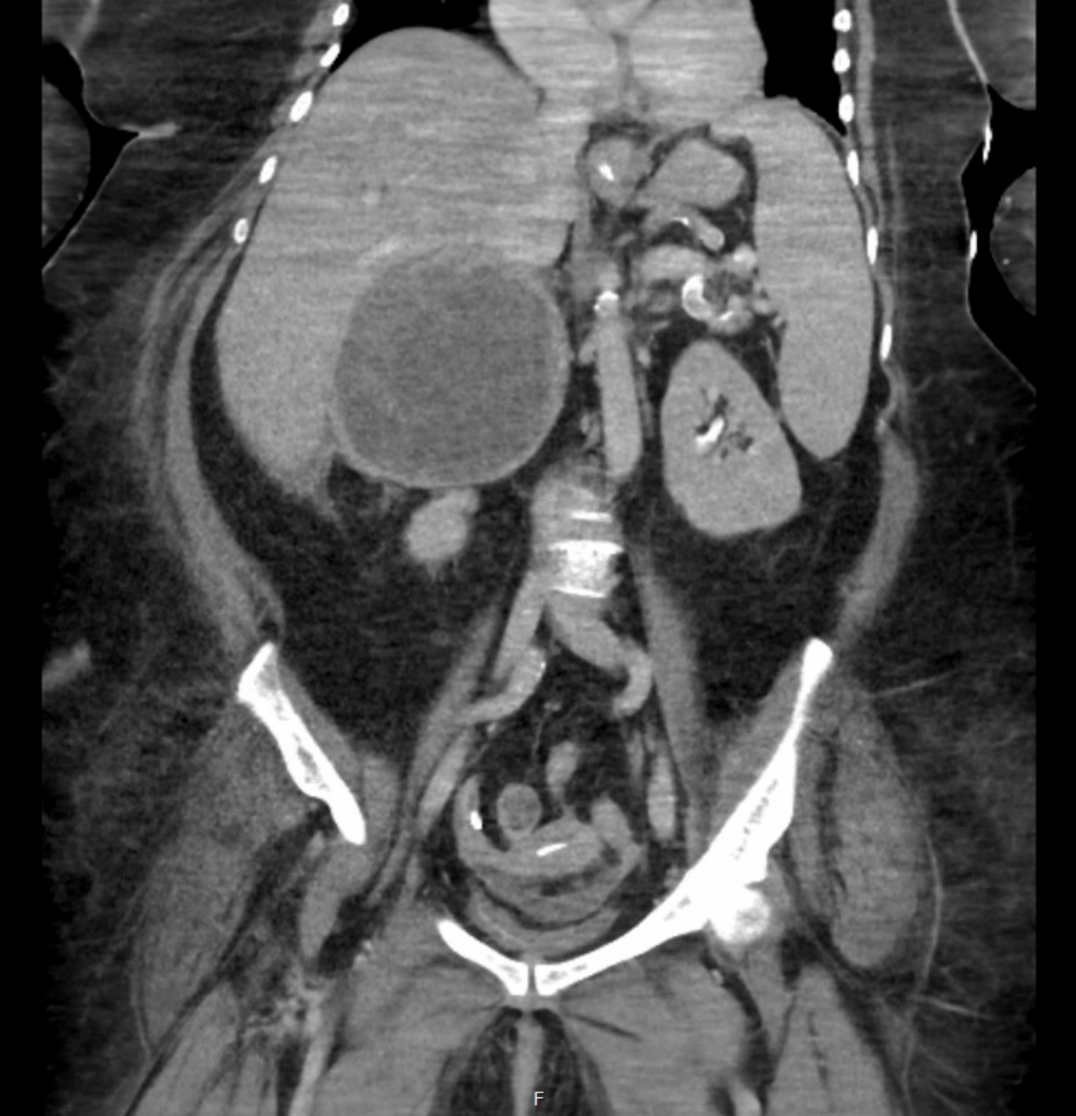
Representative coronal slide of abdominal computed tomography revealing a large right-sided adrenal abscess

Antibiotic therapy was broadened to meropenem and vancomycin, in addition to the commencement of renal replacement therapy aiming to correct the acid-base disturbance. The patient's clinical instability resulted in delays in abdominal imaging. When stabilized, CT imaging of the abdomen and the pelvis was performed on day 4 of admission, which showed a 850 mL collection in the right upper abdominal quadrant, favored to represent a right adrenal hemorrhage with possible superimposed infection (Figure 1). There was an additional right paracolic gutter fluid collection. Ultrasound-guided drainage of the adrenal collection yielded hemopurulent material which grew *C. fetus*, identified by matrix-assisted laser desorption/ionization time-of-flight mass spectrometry (MALDI-TOF) using the MALDI Biotyper® (Bruker) with a score of 2.07, and 16S ribosomal RNA polymerase chain reaction (PCR) and Sanger sequencing of a 397bp fragment completely matched *C. fetus* ATCC 27374 (Accession NR_043597) by BLAST search of the National Centre of Biotechnology Information GenBank database. The patient and her daughter gave a history of recent and regular consumption of raw lamb mince and liver, in traditional dishes “kibbeh nayyeh” and “kebda.”

Initial plans to medically manage the collection with an extended course of oral azithromycin 1 g daily were unsuccessful, with recurrence of fevers and rising inflammatory markers after four weeks of azithromycin monotherapy. Repeat abdominal imaging demonstrated interval enlargement of the adrenal collection with repeat interventional drainage yielding further hemopurulent material which later cultured an ESBL-producing *E. coli*. Meropenem was subsequently recommenced at a dose of 1 g three times a day (TDS), with the patient completing a six-week course with the aim of achieving abscess sterilization. When, upon cessation of antibiotics, there was recurrence of fever and rising inflammatory markers, the patient was transitioned to tigecycline 50 mg twice a day (BD), to additionally cover an isolated vancomycin-resistant *Enterococcus faecium* (VRE). With the aim of attaining source control, multiple surgical interventions were attempted including further radiology-guided drainage and direct abscess decortication via both a laparoscopic approach and via a pediatric endoscope advanced through a previous drain site. Unfortunately, no methods were successful, and the patient’s high anesthetic risk precluded further extensive operative management, such as laparotomy. She experienced clinical deterioration despite suppressive antibiotics, including significant bone marrow suppression, requiring regular red blood cell transfusions, and a generalized tonic-clonic seizure. Multiple repeat cultures of blood and of tissue obtained from the abdominal collection failed to identify additional pathogens, including fungal. The patient was transitioned to palliative measures on day 196 of admission; however, she was ultimately discharged to a residential aged care facility after developing a draining sinus from the abscess.

Other aspects complicating her admission included the development of an abdominal ileuspathy and diabetic neuropathy as opposed to a demyelination and a length-dependent sensorimotor peripheral neuropathy. Nerve conduction studies determined that this was most consistent with a combined critical illness neuron disorder such as Guillain-Barré syndrome. While at baseline, the patient’s function was limited to short distances of housebound mobility, because of this neuropathy, she remained bedbound throughout her admission and was unable to substantially participate in physical therapy.

## Discussion

Complications arising from *C. fetus* infections can occur from dissemination, notably endovascular infections including mycotic aneurysms with thrombosis. While the endovascular disease is well described, pure abscesses of *C. fetus* appear less commonly described; a limited number of case reports detail infections including meningitis and brain abscess, osteomyelitis with abscess formation, empyema and lung abscess, tubo-ovarian abscess, and thyroid abscess formation (Table 2) [2-7]. Older age (≥65 years), immunocompromised status, liver disease, and diabetes mellitus increase the risk of infection; however, disseminated disease has also been described in young, immunocompetent patients [1,3].

**Table 2:** Summary of available case reports of abscess-forming Campylobacter fetus (C. fetus) infections including site of infection, method of Campylobacter identification, baseline patient demographics, comorbidities, risk factors for infection identified, and treatment received MALDI-TOF: Matrix-assisted laser desorption/ionization time-of-flight mass spectrometry; IV: intravenous

Reference	*C. fetus *infection site	Method of *C. fetus* identification	Patient age	Patient sex		Patient comorbidities	Potential *C. fetus *exposure	Treatment received
Amoah et al., 2023 [2]	Cavernous sinus malformation	Culture of blood, cerebrospinal fluid, and operative samples	16		Female	Previously healthy	Livestock exposure	Surgical resection and IV meropenem for three weeks
El Beayni et al., 2020 [3]	Thyroid gland abscess	Culture of thyroid abscess aspirate. Confirmed on MALDI-TOF analysis	36		Female	Previous gastric bypass surgery	Nil	Percutaneous aspiration and intravenous amoxicillin-clavulanic acid. Duration unclear
Kitamura et al., 2016 [4]	Tubo-ovarian abscess	Culture of blood and uterine muscle fluid	28		Female	Nil reported	Recent gastrointestinal illness after barbecue where she ate cooked beef, pork, and chicken	Surgical resection and empirical cefmetazole for seven days followed by ampicillin for 14 days as per sensitivity results
Kitamura et al., 2016 [4]	Tubo-ovarian abscess	Culture of blood and operative samples	22		Female	Nil reported	Nil reported	Surgical resection followed by ceftriaxone and gentamicin for 16 days
Luo et al., 2023 [5]	Psoas abscess	Next-generation DNA and RNA sequencing of psoas abscess aspirate	66		Male	Gout	Livestock exposure	Empirical ceftriaxone and levofloxacin, followed by meropenem. Discharged on oral amoxicillin-clavulanate. Duration unclear
Targan et al., 1977 [6]	Pulmonary abscess and empyema	Culture of pleural fluid	24		Female	Polysubstance use disorder (heroin, barbiturates, diazepam)	Intravenous drug use	Empirical penicillin G and methicillin, pleural drainage with addition of gentamicin when C. fetus isolated. Total antibiotic course was 40 days
Wong et al., [7] 2009	Epidural abscess	Culture of blood and epidural abscess aspirate. Blood culture result confirmed on 16srRNA sequencing	83		Male	Ischemic heart disease, heart failure, Parkinson’s disease, chronic obstructive pulmonary disease, previous perforated duodenal ulcer	Nil identified	Surgical drainage then IV cefotaxime followed by oral ciprofloxacin until patients death on day 63

The main reservoir for *C. fetus* is the gastrointestinal tract of cattle and sheep. In a survey of sheep in New Zealand, Dempster et al. found that 48% of all sheep tested had serological evidence of previous *C. fetus* infection, and over 89% of flocks had positive antibodies to *C. fetus* [8]. *C. fetus* may spread to humans via consumption of contaminated food such as raw meat or animal liver, resulting in a mild diarrheal illness that is often self-limiting. However, certain virulence factors may aid in the development of more severe, systemic disease. Bacterial surface layer proteins (SLPs) form a capsule preventing opsonization and complement-mediated killing as well as providing antigenic variation through varying the SLP structure. These virulence factors may also explain why infections can relapse or persist in some individuals [1].

Our patient’s presentation was atypical for several reasons: firstly, for presenting with a *C. fetus* infection in profound shock; secondly, for the patient’s relative immunocompetency; and finally, owing to the finding of an adrenal abscess in the absence of concurrent bacteremia, with pure growth of *C. fetus*. The patient enjoyed eating a range of traditional raw liver and lamb mince dishes, though had not recently changed meat supplier nor had anyone else in the family been unwell. We were unable to gain further history as to the time between the purchasing of raw meat and its consumption, nor the manner or length of time taken to prepare it. These may be potential risk factors for continued inoculum of *C. fetus* and thus contribute toward the burden of disease leading to her presentation with profound shock. Despite having an adrenal abscess, her morning cortisol was not suppressed, and so adrenal suppression was considered unlikely to be contributing to shock. Of interest, this contrasts to the profound shock observed in patients with disseminated meningococcal disease who have adrenal involvement (Friedrich-Waterhouse syndrome). Additional factor that may have contributed to our patient’s severe disease phenotype was her relatively poorly controlled diabetes mellitus (glycosylated hemoglobin on admission was 8.1% (RI, 4%-6%), or 65 mmol/mol (RI, 20-42 mmol/mol). Apart from this, our patient was considered relatively immunocompetent, as they were not on any immunosuppressive medications and had an unremarkable secondary immunodeficiency screen which included testing for human immunodeficiency virus, lymphocyte subsets and immunoglobulin subsets.

There has only been one previously documented case of adrenal infection with *C. fetus*. Abe et al. described a 53-year-old female who presented with a pheochromocytoma crisis and abscess formation, whose background was notable for diabetes mellitus. Blood cultures and surgically collected adrenal tissue were positive for *C. fetus;* however, unlike our patient, the inflammatory markers were low on presentation (CRP;, 19mg/dL; RI, ≤5 mg/dL]), and their patient improved with a course of meropenem followed by ciprofloxacin [9]. In a recent systematic review of adrenal abscesses, Gligorijevic et al. found that fungal pathogens were more commonly isolated compared to bacterial pathogens, the latter of which compromised *Mycobacterium tuberculosis*, *Staphylococcus aureus*, *Streptococcus pyogenes,* or enteric pathogens. *Histoplasma capsulatum* and *M. tuberculosis* were the two most common pathogens [10].

Our patient’s clinical course was complicated by several issues, in part owing to her prolonged hospitalization and known comorbidities. A length-dependent bilateral lower limb sensorimotor neuropathy developed in the setting of her critical illness; nerve conduction studies and the clinical trajectory were not in keeping with Guillain-Barré syndrome (GBS) or acute demyelinating inflammatory polyneuropathy (AIDP). A preceding *Campylobacter *diarrheal illness is well described prior to GBS; however, it is usually *C. jejuni* or *C. coli*, and there are no clearly documented cases caused by *C. fetus* to date in the literature, though this may be due to under-recognition [11,12]. *Clostridioides difficile* infection has been described in other case reports of *C. fetus* infections, though this is presumably due to the prolonged antimicrobial therapy required for treatment rather than a direct causal relationship [7]. Additionally, our patient hemorrhaged into the abscess cavity approximately four weeks into therapy, with drain cultures subsequently detecting a pure growth of ESBL-producing *E. coli* and no growth of *C. fetus*. This may have been secondary to the development of a mycotic aneurysm that subsequently ruptured (although no aneurysm was detected on CT angiography) or a secondary bacterial infection following translocation from the gastrointestinal tract as our patient was known to be colonized with ESBL *E. coli*. As such, the six weeks of treatment (three weeks of meropenem, then three of azithromycin) appeared to be adequate to clear *C. fetus* from the abscess cavity, suggesting that azithromycin may be a reasonable option for oral stepdown therapy for susceptible *C. fetus* abscesses given its good tissue penetration and long intracellular half-life, despite the original in vitro studies that suggested a low pH environment (as in an abscess) would increase the mean inhibitory concentration and thus increase the dose required to treat an infection [13,14].

## Conclusions

Adrenal abscess is an uncommon entity to encounter in clinical practice, mostly associated with histoplasmosis or tuberculosis in patients with the appropriate epidemiological risk factors. Here, we present a rare case of adrenal abscess formation secondary to *C. fetus *infection. *C. fetus* is an uncommon but important pathogen in human disease. It has been associated with severe infectious complications including disseminated infection, endovascular involvement, and in rare cases, abscess formation. The diagnosis of *C. fetus* infection requires microbiological confirmation and should be suspected in patients presenting with bacterial infections in the context of livestock exposure or consumption of raw or undercooked meat. In the absence of defined treatment guidelines, cases of complicated *C. fetus* infection should be managed with appropriate source control and susceptibility-testing-guided antimicrobials appropriate for the site of infection. In our case, while source control was not achievable, a three-week course of meropenem followed by three weeks of azithromycin appeared to eradicate *C. fetus* from the abscess cavity.
